# Supplementary material for: Epidemiological Investigation of the First 135 COVID-19 Cases in Brunei: Implications for Surveillance, Control, and Travel Restrictions
Source: Am J Trop Med Hyg. 2020 Aug 14;103(4):1608–13. doi: 10.4269/ajtmh.20-0771 (PMC7543844; doi:10.4269/ajtmh.20-0771)
Supplement: Supplementary file 1 [file tpmd200771.SD1.pdf]

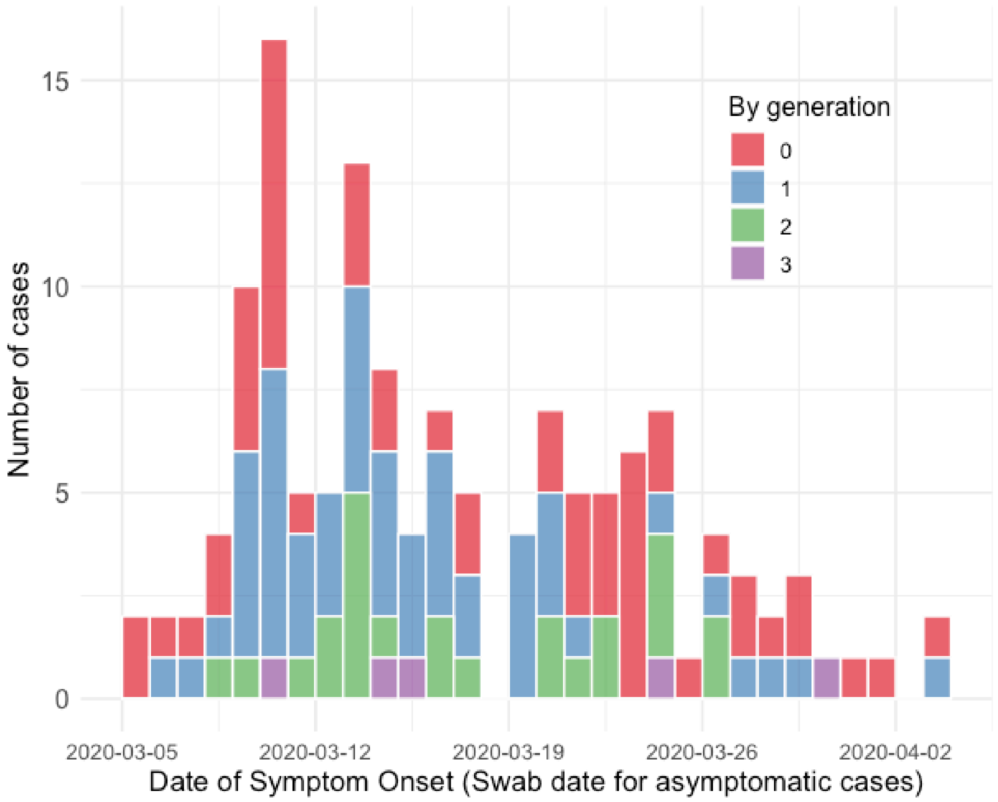

Supplemental Figure 1. Epidemic curve for the first 135 COVID-19 cases in Brunei Darussalam, by generations 0 to 3.

Note: Locally transmitted cases with known epidemiological links to an imported case were classified as generation 1 (has clear exposure to the imported cases), generation 2 (has clear exposure to generation 1 cases), and generation 3 (has clear exposure to generation 2 cases). An epidemiological link is defined as one where the local contact case has clear exposure risk to a known COVID-19 case (with correct time sequence) and had no other relevant history within the preceding 2 weeks.

Supplemental Table 1: SARS-CoV-2 testing criteria in Brunei

Version and date of implementation	SARS-CoV-2 testing criteria
V1, 23 Jan 2020	· All individuals with mild respiratory symptoms with travel history in the last 14 days to Wuhan/Hubei province or hospital contact where the disease has been reported or contact with confirmed case<br>· All individuals with moderate-severe respiratory symptoms with travel history to China in the last 14 days.
V2, 13 Feb 2020	A. Patients with severe acute respiratory infection (fever, cough, and requiring admission to hospital), AND with no other etiology that fully explains the clinical presentation AND at least one of the following:<br>· a history of travel to or residence in Mainland China, Hong Kong, Macao, Taiwan, or Singapore, in the 14 days prior to symptom onset, or<br>· patient is a health care worker who has been working in an environment where severe acute respiratory infections of unknown etiology are being cared for.<br><br>B. Patients with any acute respiratory illness AND at least one of the following:<br>· close contact with a confirmed or probable case of COVID-19 the 14 days prior to illness onset, or<br>· visiting or living in Hubei, Jiangsu or Zhejiang Province, China in the 14 days prior to symptom onset, or<br>· worked or attended a health care facility in the 14 days prior to onset of symptoms where patients with hospital-associated COVID-19 infections have been reported.
V3, 23 Feb 2020	A. All patients with severe acute respiratory infection requiring admission to hospital<br><br>B. Patients with any acute respiratory illness AND at least one of the following:<br>· close contact with a confirmed or probable case of COVID-19 the 14 days prior to illness onset, or<br>· visiting or living in any country with local transmission of SARS-CoV-2 in the 14 days prior to symptom onset
V4, 4 Mar 2020	A. All patients with severe acute respiratory infection requiring admission to hospital<br><br>B. All close contacts of confirmed cases<br><br>C. Patients with any acute respiratory illness and a travel history of visiting or living in any country with local transmission of SARS-CoV-2 in the 14 days prior to symptom onset

V5, 25 Mar 2020	A. All patients with severe acute respiratory infection requiring admission to hospital<br><br>B. All close contacts of confirmed cases<br><br>C. Patients with any acute respiratory illness and a travel history of visiting or living in any country with local transmission of SARS-CoV-2 in the 14 days prior to symptom onset<br><br>D. Patients presenting with respiratory symptoms for the second time within the last fourteen days

Supplemental Table 2. Symptoms presented at presentation and during admission, among the first 135 COVID-19 cases in Brunei Darussalam

Reported symptoms		n (%)
**Respiratory**	Fever	84 (62.2)
	Cough	80 (59.3)
	Runny nose	41 (30.4)
	Sneezing	4 (3.0)
	Sore throat	84 (62.2)
	Shortness of breath	14 (10.4)
**General**	Body/muscle ache	24 (17.8)
	Joint Ache	7 (5.2)
	Chills	16 (11.9)
	Headache	29 (21.5)
	Loss of smell	2 (1.5)
	Fatigue	19 (14.1)
	Insomnia	5 (3.7)
**Gastro-intestional**	Nausea	12 (8.9)
	Vomiting	14 (10.4)
	Loss of Appetite	24 (17.8)
	Dyspepsia	8 (5.9)
	Diarrhoea	41 (30.4)
